# A WEE1 family business: regulation of mitosis, cancer progression, and therapeutic target

**DOI:** 10.1186/s13045-020-00959-2

**Published:** 2020-09-21

**Authors:** Andrea Ghelli Luserna di Rorà, Claudio Cerchione, Giovanni Martinelli, Giorgia Simonetti

**Affiliations:** Biosciences Laboratory (Onco-hematology Unit), Istituto Scientifico Romagnolo per lo Studio e la Cura dei Tumori (IRST) IRCCS, Via P. Maroncelli 40, 47014 Meldola, FC Italy

**Keywords:** WEE1 family kinases, WEE1, PKMYT1, Cell cycle, DNA repair, Pseudo-oncogene, Tumor suppressor

## Abstract

The inhibition of the DNA damage response (DDR) pathway in the treatment of cancer has recently gained interest, and different DDR inhibitors have been developed. Among them, the most promising ones target the WEE1 kinase family, which has a crucial role in cell cycle regulation and DNA damage identification and repair in both nonmalignant and cancer cells. This review recapitulates and discusses the most recent findings on the biological function of WEE1/PKMYT1 during the cell cycle and in the DNA damage repair, with a focus on their dual role as tumor suppressors in nonmalignant cells and pseudo-oncogenes in cancer cells. We here report the available data on the molecular and functional alterations of WEE1/PKMYT1 kinases in both hematological and solid tumors. Moreover, we summarize the preclinical information on 36 chemo/radiotherapy agents, and in particular their effect on cell cycle checkpoints and on the cellular WEE1/PKMYT1-dependent response. Finally, this review outlines the most important pre-clinical and clinical data available on the efficacy of WEE1/PKMYT1 inhibitors in monotherapy and in combination with chemo/radiotherapy agents or with other selective inhibitors currently used or under evaluation for the treatment of cancer patients.

## Background

The WEE1 kinase family consists of three serine/threonine kinases sharing conserved molecular structures and encoded by the following genes: *WEE1* (WEE1 G2 Checkpoint Kinase), *PKMYT1* (membrane-associated tyrosine- and threonine-specific cdc2-inhibitory kinase), and *WEE1B* (WEE2 oocyte meiosis inhibiting kinase). In eukaryotic somatic cells, WEE1 and PKMYT1 play a key role in cell cycle regulation and, in particular, they are involved in the entry into mitosis [1]. Their role as regulators is crucial during normal cell cycle progression and in response to DNA damages, as part of the DNA damage response (DDR) pathways. Similarly, WEE2 regulates cell cycle progression and, in particular, meiosis [2]. Briefly, WEE2 plays a dual regulatory role in oocyte meiosis by preventing premature restart prior to ovulation and permitting metaphase II exit at fertilization [3]. Despite the identification of WEE2 somatic mutations (1.9% of cases) and copy number (CN) alterations (22.5% of patients with CN loss and 22.5% with CN gain) across several cancer types (https://portal.gdc.cancer.gov), they have not been functionally linked to tumor development so far. Therefore, the following sections will be focused on WEE1 and PKMYT1 kinases that have a well-recognized role in oncology and hemato-oncology.

## WEE1 and PKMYT1 in cell cycle regulation

**Fig. 1:**
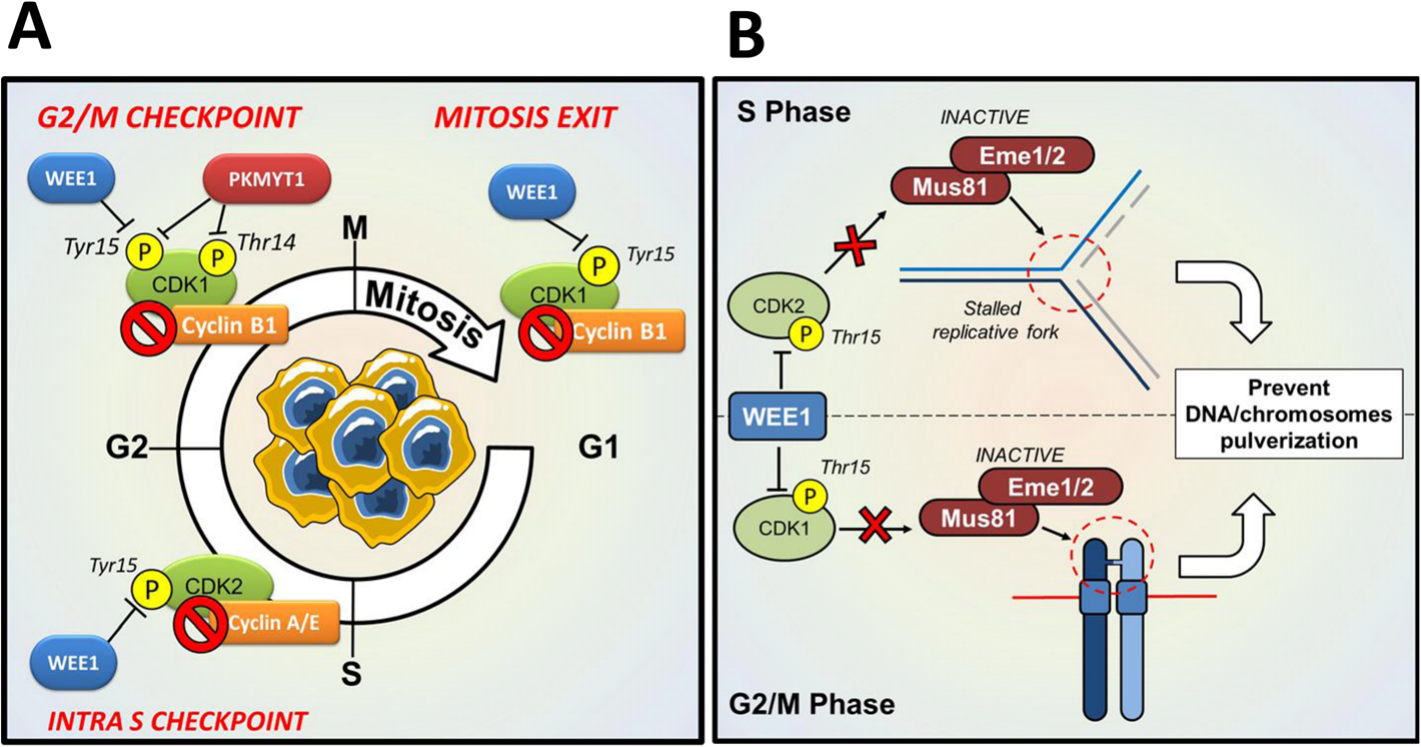
WEE1 and PKMYT1 biological functions. **a** Schematic representation of WEE1 and PKMYT1 involvement in cell cycle checkpoints. WEE1 regulates the activity of both CDK1 and CDK2 kinases (trough phosphorylation of Tyr15) and is involved in the regulation of intra-S, G2/M, and M phase cell cycle checkpoints. PKMYT1 selectively regulates CDK1 (through phosphorylation of Tyr15 and Thr14) and is plays a role in the G2/M phase checkpoint. **b** Schematic representation of the regulation of MUS81-EME1/2 endonuclease complexes by WEE1 during S and G2/M cell cycle phases. By inhibiting CDK2 or CDK1, WEE1 prevents MUS81 activation and the generation of DNA damages during S phase, and chromosomes pulverization during G2/M phase

WEE1 and PKMYT1 act as tumor suppressors in non-malignant eukaryotic somatic cells. Similarly to other DDR-related kinases, their main biological function is to prevent replication of cells with altered DNA. The main downstream target of WEE1 family kinases is the cyclin-dependent kinase 1 (CDK1)-cyclin B1 complex, also known as mitotic-promoting factor (MPF). WEE1 phosphorylates CDK1 on Tyr15 while PKMYT1 has a dual activity on Tyr15 and Thr14 [4] (Fig. 1a). The phosphorylation of those residues keeps the MPF complex inhibited until the cell approaches mitosis. WEE1 is located in the nucleus, while PKMYT1 is associated with the endoplasmic reticulum and Golgi apparatus [5, 6], and regulates Golgi membrane reassembly following mitosis [7]. Together, WEE1 and PKMYT1 ensure that CDK1 remains inactive as it shuttles into and out of the nucleus [8]. Through its extra-nuclear localization, PKMYT1 can also promote CDK1 cytosolic segregation. At the G2/M border, if no DNA damage has been detected, CDK1 phosphorylation on Tyr15 and Thr14 is rapidly removed by CDC25C phosphatase. In the nucleus, the CDK-activating kinase (CAK) complex composed by cyclin-dependent kinase 7 (CDK7), cyclin H1, and MAT1 promotes MPF complex activation through the phosphorylation of CDK1(Thr161) [9, 10]. The active MPF complex is then imported into the nucleus through phosphorylation of cyclin B1 (Ser126, Ser128, Ser133, and Ser147) [11]. This event is required to enter mitosis. The relevance of WEE1 and PKMYT1 regulation of CDK1 has been recently confirmed by in vivo studies. Indeed, the replacement of the CDK1 inhibitory phosphorylation sites with non-phosphorylatable amino acids (CDK1^T14A/Y15F^) was embryonic lethal in mice [12]. Once activated, the MPF complex can phosphorylate WEE1 and PKMYT1 to promote their inactivation via different cascades [5, 13, 14]. WEE1 is phosphorylated (Ser123) by CDK1 at the onset of mitosis, thereby generating a binding motif for polo like kinase 1 (PLK1) and casein kinase 2 (CK2), that in turn phosphorylate WEE1 (Ser53 and Ser121, respectively) [14, 15]. Together, the phosphorylation of the three Ser residues serves as a tag for the degradation of WEE1 by the ubiquitin ligase SCFβ-TrCP [13]. PKMYT1 is also phosphorylated by CDK1 and PLK1 and this event promotes its degradation [16]. In addition to the checkpoint function at the G2/M border, recent findings highlighted a role of WEE1 in the regulation of replication dynamics during S phase (intra S phase checkpoint). When cells reach the S phase, replication is initiated from a large number of replication origins triggered through the activation of the pre-replication complex [17] and following the activation of S phase specific CDK, primarily CDK2 [18, 19]. Similarly to CDK1, CDK2 regulation is controlled through Tyr15 phosphorylation status, that is balanced by WEE1 (Fig. 1a) and cell division cycle 25A (CDC25A) activity [20]. Both WEE1 and CDC25A/C have been shown to modulate unperturbed replication through regulating CDK1/CDK2 activity. Monoallelic expression of CDK1^T14A/Y15F^ induced replication stress and S phase arrest in mouse embryonic fibroblasts (MEFs), with substantial increase of γH2AX levels, chromosomal fragmentation, and DDR activation, as a consequence of intra-S phase DNA damage [12]. Moreover, unscheduled origin firing due to loss of WEE1 leads to exhaustion of the replication protein A1 (RPA1) pool and, as a consequence, to death during DNA replication (replication catastrophe). The intra S phase activity of WEE1 is independent from PKMYT1 that is unable to phosphorylate CDK2 [5]. In addition, WEE1, but not PKMYT1, contributes to the control of mitosis exit. Indeed, *Wee1*-deficient MEFs showed mitotic defects (e.g., in the number and position of centrosomes) that induce arrest in mitosis or, in the majority of cells, mitotic slippage [21, 22]. At the end of mitosis, WEE1 inhibits CDK1 through phosphorylation of its Tyr15 residue (Fig. 1a). This event is dependent on the activation of the CTD phosphatase subunit 1 (FCP1) that dephosphorylates and activates WEE1 and other crucial component of the spindle assembly checkpoint (SAC) complex [23]. Although the precise mechanisms that regulate FCP1 activity is still unknown, it has been showed that FCP1 promotes the dephosphorylation of crucial SAC components, including cell division cycle 20 (CDC20) and ubiquitin specific peptidase 44 (USP44), thus promoting APC/C^Cdc20^ activation and chromosome segregation [24–26]. Moreover, WEE1 directly interacts with APC/C components, including fizzy and cell division cycle 20 related 1 (CDH1), CDC20, cell division cycle 27 (CDC27), and its deletion enforced APC/C activity, resulting in alterations of the level of APC/C substrates and mitosis progression at the expense of genomic stability [21].

## WEE1 regulates replication forks and genome stability

The activity of WEE1 through the cell cycle can explain its tumor suppressor function, at least in nonmalignant cells. This observation was confirmed and disentangled in preclinical studies. Indeed, conditional *Wee1* heterozygous deletion in the murine mammary epithelium caused enhanced proliferation, with cells progressing into mitosis while still undergoing DNA replication, and consequent accumulation of DNA damage, resulting in genomic instability and, ultimately, in tumor development [21]. Biological processes such as DNA replication and homologous recombination involve the formation of branched DNA structures that physically link chromosomes. Such DNA structures needs to be disengaged prior to entry into mitosis, in order to ensure proper chromosome segregation. Eukaryotic cells evolved different mechanisms to identify and process branched DNA structures (Y-shape DNA) and the most important one involves the structure-selective endonuclease MUS81. MUS81 forms heterodimeric complexes with the non-catalytic subunits EME1 or EME2 and recognizes Y-shape DNA structures during DNA replication or during mitosis (homologous recombination). The activity of MUS81-EME1/2 complex is crucial to recover stalled replication forks, during prolonged S phase arrest, and to reset DNA junction between twin chromatids during homologous recombination [27]. In unperturbed cells, WEE1 protects replication forks and prevents the generation DNA damages and chromosome pulverization through an indirect inhibition of MUS81 functionality [28]. Indeed, WEE1 phosphorylates CDK1 and CDK2, thus preventing the CDK-mediated phosphorylation and activation of MUS81-EME1/2 complexes [29]. Lack of WEE1-dependent regulation of MUS81-EME1/2 endonucleases may lead to cleavage of unwanted DNA structure (excessive replication forks), which would slow down replication progression and increase genomic instability [27, 28] (Fig. 1b).

## WEE1 and PKMYT1 deregulation in cancer cells

### WEE1 and PKMYT1 act like oncogenes

**Fig. 2:**
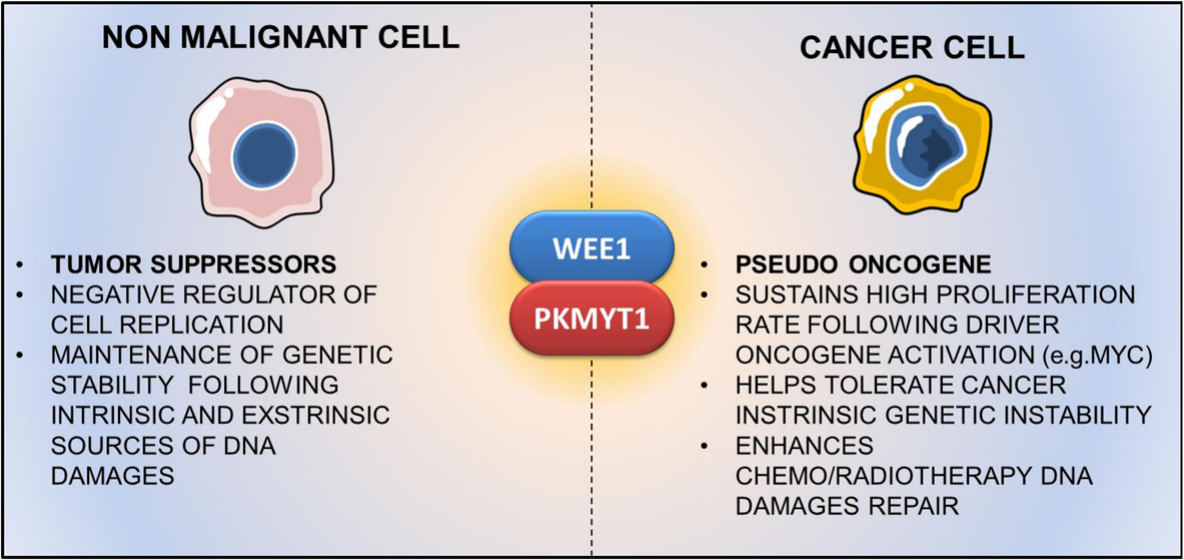
WEE1 family proteins role as tumor suppressors or pseudo-oncogenes in non-malignant and cancer cells

The biological role of WEE1 and PKMYT1 in cancer cells is not fully understood. Reduced WEE1 expression has been detected in breast cancer compared with normal tissues, independently of the tumor grade [21]. However, most findings suggest that both kinases act like oncogenes rather than tumor suppressors. Indeed, they are frequently overexpressed in both solid and hematological tumors and a genome-wide CRISPR screen of 563 cancer cell lines, showed that they are essential for the cell viability of almost all cell lines [30]. The dependency of cancer cells on WEE1 family proteins may be linked to the following mechanisms (Fig. 2): (i) the high proliferation rate of cancer cells that follows the activation of driver oncogenes (e.g. RAS, MYC) needs to be sustained by a strong cell cycle regulation machinery; (ii) cancer cells frequently inactivate p53, which is a key gatekeeper of G0/G1 and S phases and, as a consequence, the regulation of cell cycle is sustained entirely by the G2/M checkpoint; (iii) the over-expression of DDR-related kinases is fundamental to maintain a tolerable level of genetic instability, an intrinsic feature of cancer cells [31, 32]. Therefore, we can speculate that, once the malignant transformation process has been induced, WEE1 upregulation exerts a pro-tumorigenic functions by securing a tolerable level of genomic instability to cancer cells. The following sections summarize the current knowledge on the molecular and functional alterations of WEE1 and PKMYT1 in hematological and solid tumors.

### WEE1 and PKMYT1 genetic lesions in cancer

**Fig. 3:**
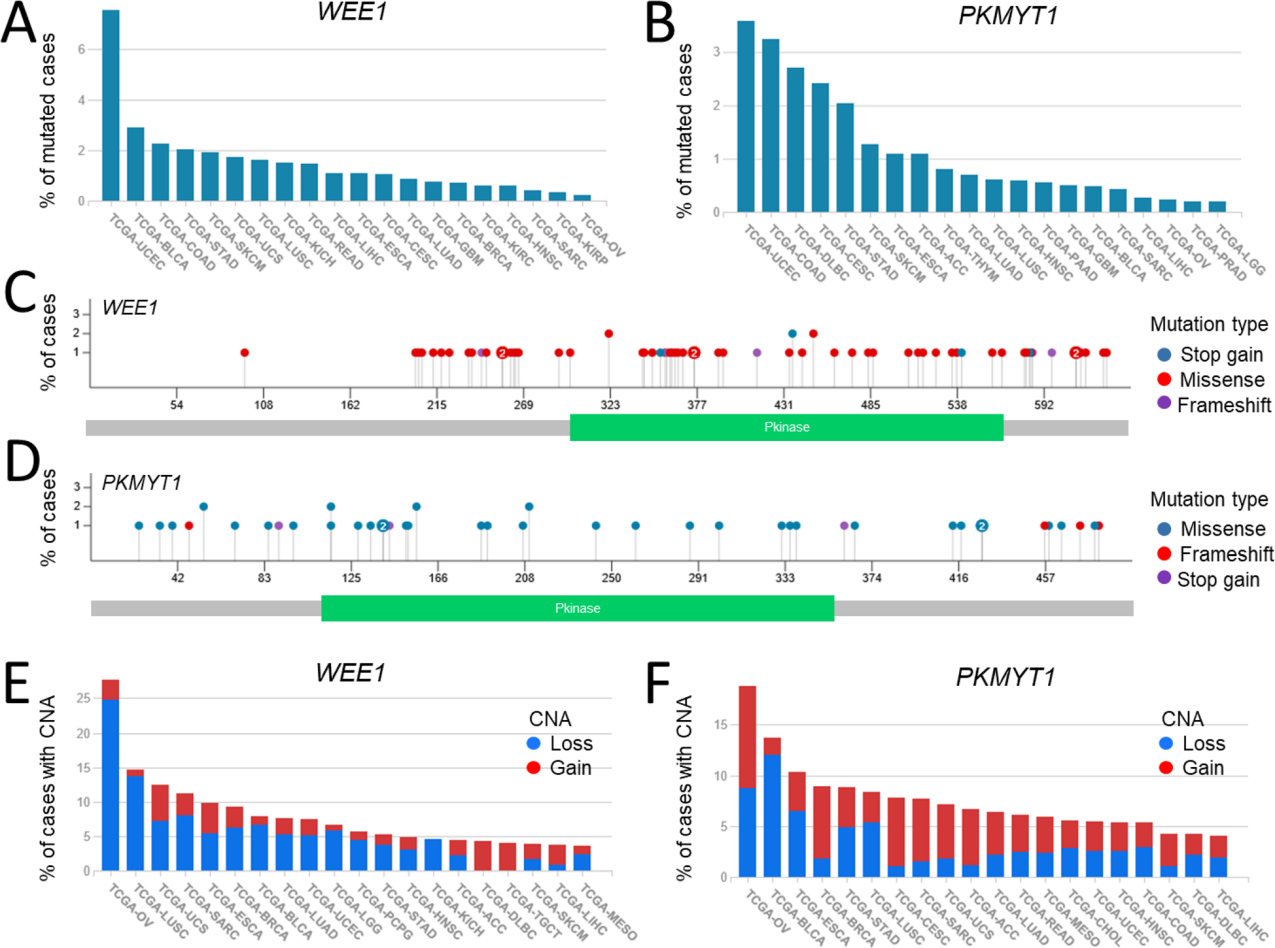
*WEE1* and *PKMYT1* mutations and copy number alterations (CNAs) in cancer. **a** Frequency of patients with *WEE1* or **b** *PKMYT1* gene mutations across cancers from TCGA cohorts. **c** Distribution of mutations according to the WEE1 and **d** PKMYT1 amino acid (aa) sequence and protein domains (WEE1 transcript ENST00000450114, 646 aa; PKMYT1 transcript ENST00000262300, 499 aa). **e** Frequency of patients with copy number gain or loss in *WEE1* or **f** *PKMYT1* across cancers (https://portal.gdc.cancer.gov; *ACC* adrenocortical carcinoma, *BLCA* bladder urothelial carcinoma, *BRCA* breast invasive carcinoma, *CESC* cervical squamous cell carcinoma and endocervical adenocarcinoma, *COAD* colon adenocarcinoma, *CHOL* cholangiocarcinoma, *DLBC* diffuse large B cell lymphoma, *ESCA* esophageal carcinoma, *GBM* glioblastoma multiforme, *HNSC* head and neck squamous cell carcinoma, *KICH* kidney chromophobe, *KIRK* kidney renal clear cell carcinoma, *KIRP* kidney renal papillary cell carcinoma, *LGG* brain lower grade glioma, *LIHC* liver hepatocellular carcinoma, *LUAD* lung adenocarcinoma, *LUSC* lung squamous cell carcinoma, *MESO* mesothelioma, *OV* ovarian serous cystadenocarcinoma, *PAAD* pancreatic adenocarcinoma, *PCPG* pheochromocytoma and paraganglioma, *PRAD* prostate adenocarcinoma, *READ* rectum adenocarcinoma, *SARC* sarcoma, *SKCM* skin cutaneous melanoma, *STAD* stomach adenocarcinoma, *TGCT* testicular germ cell tumors, *THYM* thymoma, *UCS* uterine carcinosarcoma, *UCEC* uterine corpus endometrial carcinoma)

*WEE1* and *PKMYT1* are rarely mutated in cancer patients, with an overall mutation frequency of 1.2% and 0.2%, respectively (https://portal.gdc.cancer.gov). The distribution of somatic mutations is highly heterogeneous across cancer types (*WEE1*: 0.2–7.6%; *PKMYT1*:0.1–3.6%), with a higher frequency in uterine corpus endometrial carcinoma (UCEC) and tumors of the gastrointestinal tract (stomach and colon adenocarcinoma, Fig. 3a, b). In particular, *WEE1* mutations have been reported in 7.6% of UCEC cases. Moreover, *PKMYT1* lesions have been also detected in 2.7% of diffuse large B cell lymphoma (DLBC). Conversely, both kinase genes are rarely mutated in brain lower grade glioma (LGG), ovarian serous cystadenocarcinoma (OV), prostate adenocarcinoma (PRAD), and sarcoma (SARC), with a frequency lower than 0.5%. Both genes are mainly targeted by missense mutations that preferentially cluster in the region encoding the *WEE1* kinase domain and its surroundings (Fig. 3c), suggesting a potential gain of function effect of the kinase activity. Conversely, the mutations are scattered throughout the *PKMYT1* sequence (Fig. 3d). Little is known about the functional consequences of *WEE1* and *PKMYT1* mutations. In the majority of cancer types, the transcript expression in the mutated cases is higher than the median value of the entire cohort (https://www.cbioportal.org), supporting once more an oncogenic function. In pancreatic adenocarcinoma (PA) patients and cell lines, an insertion was identified in the *WEE1* poly-T track, which contains the binding site of the HuR RNA binding protein [33]. The insertion resulted in decreased WEE1 expression upon mitomycin-induced DNA damage, which would argue against a protective effect of the mutation. Copy number alterations (CNAs) represent a more frequent event compared with mutations, with the *WEE1* gene being predominantly involved in CN loss (23.7% of cases versus 7.8% of patients with CN gains), while *PKMYT1* showing a higher percentage of CN gain (15.9% versus 12.0% of CN loss, Fig. 3e, f). The predominance of *WEE1* deletion events (6.3% versus 3.25% of cases with amplification) was also observed in breast cancer, in line with its reduced expression, as mentioned above [21]. Overall, cancer types showing the highest recurrence (> 10%) of CNAs were OV (27.7%), lung squamous cell carcinoma (LUSC, 14.8%), uterine carcinosarcoma (UCS, 12.5%), and SARC (11.2%) for *WEE1* and OV (18.8%), bladder urothelial carcinoma (BLCA, 13.7%), and esophageal carcinoma (ESCA, 10.3%) for *PKMYT1*. Of note, OV and LUSC have been classified as tumors with multiple recurrent chromosomal gains and losses [34], which may suggest a bystander effect related to chromosomal instability in these tumor types, especially in the case of *WEE1* deletion, that is unexpected, based on the general oncogenic function exerted by the kinase.

### WEE1 and PKMYT1 functional role in hematological and solid tumors

Few studies have analyzed WEE1 and PKMYT1 expression in hematological malignancies. Our group showed that *WEE1* kinase is highly expressed in acute lymphoblastic leukemia (ALL) cell lines and primary cells in comparison with normal hematopoietic cells, and that *PKMYT1* is upregulated in relapsed ALL samples compared with nonmalignant hematopoietic cells [35]. Moreover, we demonstrated that ALL cells are dependent on WEE1 functionality for their survival and proliferation and that *PKMYT1* levels may influence the sensitivity to the WEE1 inhibitor AZD-1775 [35]. Similar results on the role of WEE1 were obtained in multiple myeloma (MM), acute myeloid leukemia (AML), chronic myeloid leukemia (CML), and chronic lymphocyte leukemia (CLL) [36–39]. In AML cells, *WEE1* and *PKMYT1* are key gene discriminating between *FLT3*-ITD, *FLT3*-TKD, and *NRAS*-mutated samples. They were expressed at lower levels selectively in *FLT3*-ITD specimens in comparison with wild-type cells, suggesting either a tumor suppressor role in the leukemogenic process or a potential vulnerability n this AML subtype [40]. Pharmacological WEE1 inhibition alone or in combination with histone deacetylase inhibitors showed therapeutic potential in *FLT3*-ITD AML, confirming their dependency on WEE1 activity [41]. Since *FLT3*-ITD AML have intrinsic homologous recombination repair defects [42]. WEE1 inhibition may exacerbate the cell genotoxic stress by disrupting multiple cell cycle checkpoints. WEE1 has been showed to be a valuable target also for lymphoma patients [43]. In parallel, PKMYT1 proved to be essential for MM cell line viability, since its downregulation strongly decreased cell growth, while inducing apoptosis [44].

WEE1 and PKMYT1 are also over-expressed in solid tumors, including hepatocellular carcinoma, colon cancer, glioblastoma, non-small-cell lung cancer (NSCLS), neuroblastoma, and gastric cancers [31, 32, 45–47]. High WEE1 expression has been associated with negative prognostic factors including lymph node involvement, induction of metastasis, increased biomarkers of proliferation (CCND1, Ki67, or CCNA1) and resistance to treatments (radiotherapy or chemotherapy) [48–51]. Elevated PKMYT1 levels have been associated with tumor progression, a more aggressive disease, the induction of metastasis at least in NSCLS patients [45] and, generally, with poor prognosis. Depending on the cancer subtype, the expression of WEE1 and PKMYT1 has been linked with the activation of cellular pathways crucial for the specific disease. In melanoma cells, WEE1 silencing caused an increase of phospho p38 protein levels, indicating a role in the regulation of p38/MAPK pathway activation during p53-independent DNA damage response [49]. In hepatocellular carcinoma and colorectal cancers, PKMYT1 regulates epithelial-mesenchymal transition (EMT), a process relevant to tumor progression, invasion, metastasis, and drug resistance, through the activation of the beta-catenin/TCF signaling [32, 46], while PKMYT1 has been reported to control Notch pathway in NSCLC [45]. In particular, crucial component of the pathway, including NOTCH1, p21, and HES1 are downregulated by chemical inhibition of PKMYT1 [45]. In neuroblastic tumors, PKMYT1 is required to stabilize MYCN protein, which is a crucial proto-oncogene for this cancer types [52]. Moreover, in esophageal squamous cell carcinoma (ESCC) cell lines and primary cells, the expression of PKMYT1 is associated with and regulates the activation of the AKY/mTOR pathway [53] (Table 1). Taken together, this evidence suggests a broad role of WEE1/PKMYT1 besides the DNA damage response pathway that may increase the interest towards its therapeutic targeting.

**Table 1:** *WEE1* and *PKMYT1* molecular alterations in hematological and solid tumors according to literature

Gene	Genetic alteration	Disease	Effect/prognostic value	Reference
Hematological tumors				
*WEE1*	Over-expression	ALL; AML; MM; CML; CLL; DLBCL	Crucial for cell viability of cancer cells (experimentally proven).	[35–40, 43, 54]
	Copy number Gain	AML	Biological effect or prognostic value unknown	[55]
*PKMYT1*	Over-expression	ALL; MM	Crucial for cell viability of cancer cells (experimentally proven).	[35, 44]
Solid tumors				
*WEE1*	Over-expression	GC; MaM; GL; OC; CC	Associated with lymph node involvement, induction of metastasis, increased biomarkers of proliferation (CCND1, Ki67 or CCNA1), resistance to treatment and poor overall survival.	[48–51, 56–60]
	Mutation	PA	Insertion causing decrease WEE1 expression upon DNA damage	[33]
*PKMYT1*	Over-expression	HC; CC; GLB; NSCLC; N; GS	Associated with tumor progression, aggressive disease and poor overall survival.	[31, 32, 45–47]
	Mutation	N	Biological effect or prognostic value unknown	[61]

*ALL* acute lymphoblastic leukemia, *AML* acute myeloid leukemia, *MM* multiple myeloma, *CML* chronic myeloid leukemia, *CLL* chronic lymphocyte leukemia, *DLBCL* diffuse large B cell lymphoma, *GC* gastric cancer, *MaM* malignant melanoma, *GL* gliomas, *OC* ovarian cancer, *CC* colorectal cancer, *PA* pancreatic adenocarcinoma, *HC* hepatocellular carcinoma, *GLB* glioblastoma, *NSCLC* non-small-cell lung cancer, *N* neuroblastoma

## Development of WEE1 and PKMYT1 inhibitors

### WEE1 and PKMYT1 inhibitors have single agent and chemo-sensitizer effects

**Fig. 4:**
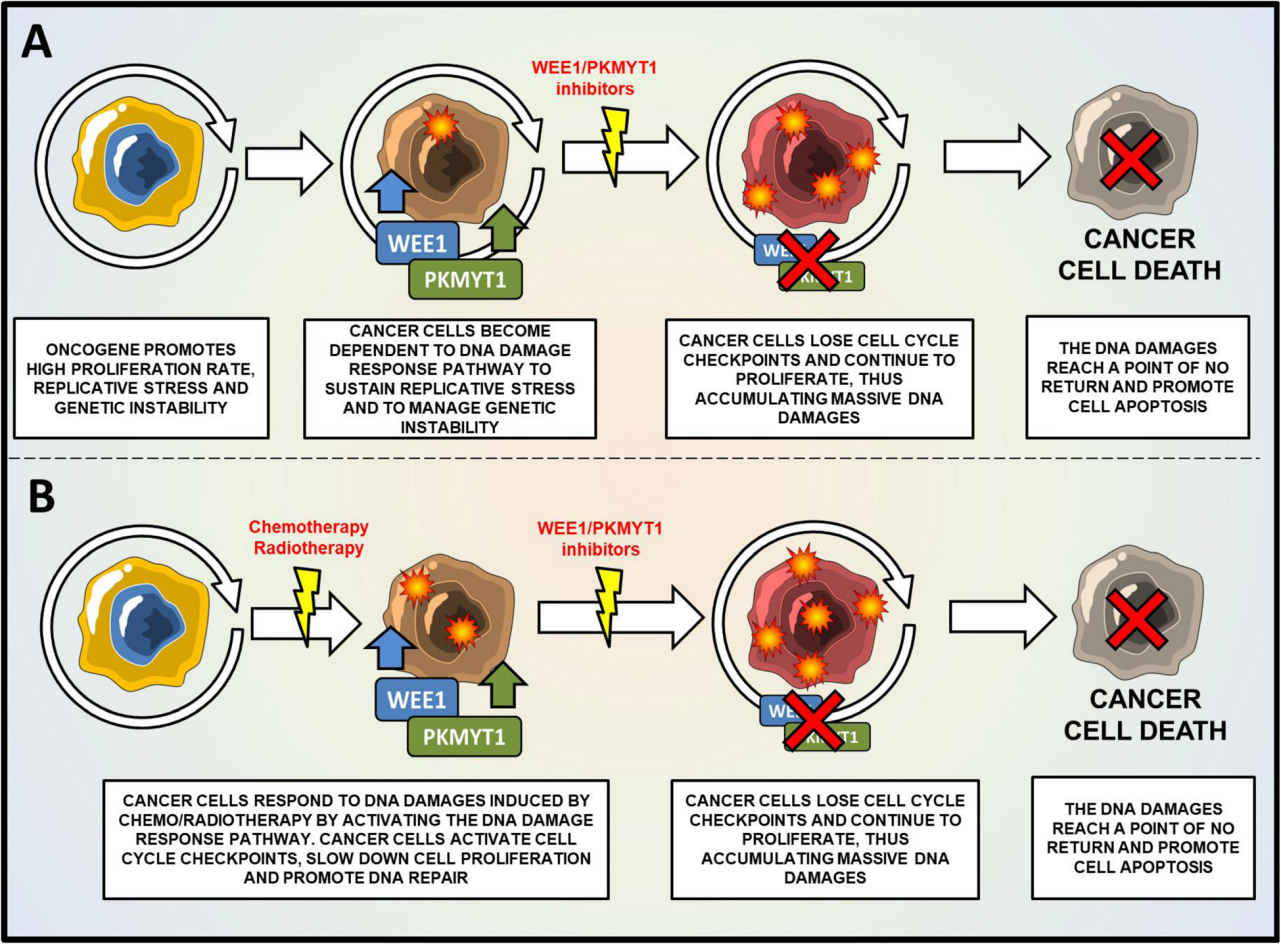
Mechanism of action of WEE1/PKMYT1 inhibitors for the treatment of cancer cells. **a** Schematic representation of WEE1/PKMYT1 inhibition as monotherapy. In cancer cells, oncogenes promote high rate of proliferation, replication stress and the over-expression of WEE1/PKMYT1 kinases. In this scenario, cancer cells need WEE1 and PKMYT1 to sustain replication stress and proliferation. The inhibition of WEE1/PKMYT1 results in the accumulation of DNA damages, the increase of genetic instability and induction of apoptosis. **b** Schematic representation of WEE1/PKMYT1 inhibition in combination with DNA damaging agents. Cancer cells respond to DNA damages by activating WEE1/PKMYT1 kinases. The inhibition of WEE1/PKMYT1 enhances the cytotoxicity of DNA damaging agents by inhibiting DNA repair and promoting cell cycle progression even in the presence of DNA damages. Therefore, cancer cells accumulate massive DNA damages until a point of no return

Due to their potential oncogenic role, WEE1 and PKMYT1 have been investigated as therapeutic targets for hematological and solid tumors. Several pharmacological inhibitors have been designed and subsequently validated in different cancer models. The available literature highlights a common mechanism of action of WEE1/PKMYT1 inhibitors in cancer cells either in single agent or in combination with DNA damaging agents (chemotherapy/radiotherapy). WEE1/PKMYT1 kinase inhibition causes G2/M cell cycle checkpoint override, premature mitotic entry, and cell death during mitosis, through a mechanism generally known as mitotic catastrophe (Fig. 4a). From a biological point of view, the inhibition of WEE1 kinase causes a significant reduction of phospho-CDK1 (Tyr15), thus promoting the accumulation of active CDK1-cyclin B1 complex and, consequently, mitotic entry. The beginning of mitosis is also associated with a progressive accumulation of DNA damages and the degeneration in mitotic catastrophe. The sensitivity to WEE1 kinase inhibitors in relation to *TP53* mutational status remains controversial. Indeed, some studies reported increased sensitivity of *TP53* mutant cell lines to WEE1 inhibitors in comparison to *TP53* wild-type ones [62, 63], while others showed no association between p53 functionality and the effectiveness of WEE1 inhibition [35, 64]. These discrepancies may be linked to the intrinsic chromosomal instability of the analyzed tumors and to additional alterations deregulating the G1 checkpoint in *TP53* wild-type cases that may enhance the sensitivity to WEE1 targeting.

Regarding the role of WEE1 inhibitors as chemo-sensitizer agents, a large number of studies demonstrated a synergistic activity between DNA damaging agents (chemotherapy including doxorubicin, cytarabine, methotrexate, cisplatin, clofarabine, etoposide, 5-fluorouracil, and radiotherapy) and different WEE1/PKMYT1 inhibitors in preclinical models [48, 56, 65–69]. The mechanism of action of the combination is based on the inhibition of the DDR pathway following induction of DNA damage induced by the chemotherapy or radiotherapy agents. In this scenario, cancer cells with damaged DNA fail to arrest cell cycle, continue to proliferate, and accumulate massive DNA damage until a point of no return (Fig. 4b). Indeed, several DNA damaging agent promote the indirect activation of WEE1 and PKMYT1 kinases, as showed mostly by the activation of cell cycle checkpoints (S and G2/M checkpoints) in cancer cells. We summarized in Table 2 the results of preclinical studies in which the effect of different chemotherapy agents or radiotherapy has been evaluated in terms of cell cycle perturbation and altered expression of WEE1 or PKMYT1 following in vitro or in vivo treatment. Taken together, the abovementioned data prove that WEE1 and PKYMYT1 are ideal targets to override cell cycle checkpoint regulation and to improve the efficacy of DNA-damaging agents. In particular, tumors with a high level of chromosomal instability may respond to WEE1/PKMYT1 inhibition per se, while cases with a more stable genomic asset may benefit of the combination between DNA-damaging agents and WEE1 family kinase inhibitors. The following sections reports the main preclinical and clinical findings obtained using small molecules inhibitors of WEE1 and PKMYT1 kinases.

**Table 2:** Effects of standard of care chemo/radiotherapy agents on cell cycle checkpoints activation

Chemotherapy agents/radiotherapy	Intra S checkpoint	G2/M checkpoint	WEE1 and/or PKMYT1 experimentally proven involvement in cancer model
*Actinomycin*	No	Yes [70, 71]	WEE1 upregulation [71]
*Azacitidine*	No	Yes [72]	NA
*Bleomycin*	No	Yes [73]	NA
*Carboplatin*	No	Yes [74]	NA
*Cisplatin*	No	Yes [75, 76]	WEE1 inhibition enhanced cytotoxicity [76, 77]
*Cyclophosphamide*	NA	NA	WEE1 upregulation [78]
*Cytarabine*	Yes [79, 80]	Yes [79, 80]	WEE1 upregulation [38, 81]
*Clofarabine*	Yes [35]	No	WEE1 inhibition enhanced cytotoxicity [35]
*Daunorubicin*	Yes [82]	Yes [82]	NA
*Decitabine*	No	Yes [83]	NA
*Docetaxel*	No	Yes [84, 85]	NA
*Doxorubicin*	No	Yes [86]	WEE1 upregulation [86]; WEE1 inhibition enhanced cytotoxicity [35]
*Epirubicin*	No	Yes [87, 88]	WEE1 inhibition enhanced cytotoxicity [89]
*Epothilone*	No	Yes [90]	NA
*Etoposide*	No	Yes [91]	WEE1 inhibition enhanced cytotoxicity [67]
*Fluorouracil*	Yes [92]	No	WEE1 inhibition enhanced cytotoxicity [92]
*Fludarabine*	Yes [80]	No	NA
*Gemcitabine*	Yes [80]	No	WEE1 upregulation [93]; WEE1 inhibition enhanced cytotoxicity [94]
*Hydroxyurea*	Yes [95]	No	WEE1 inhibition enhanced cytotoxicity [96]
*Idarubicin*	No	Yes [97]	NA
*Irinotecan*	No	Yes [98]	WEE1 inhibition enhanced cytotoxicity [99]
*Mechlorethamine*	Yes [100]	Yes [100]	NA
*Mercaptopurine*	Yes [101]	No	NA
*Methotrexate*	Yes [102]	No	WEE1 inhibition enhanced cytotoxicity [103]
*Mitoxantrone*	No	Yes [104]	WEE1 inhibition enhanced cytotoxicity [78]
*Oxaliplatin*	No	Yes [105]	WEE1 inhibition enhanced cytotoxicity [106]
*Paclitaxel*	No	Yes [107]	WEE1 inhibition enhanced cytotoxicity [108]
*Pemetrexed*	Yes [109]	No	WEE1 inhibition enhanced cytotoxicity [110]
*Radiotherapy (ionizing radiation)*	No	Yes [111]	WEE1 inhibition enhanced cytotoxicity [68]
*Teniposide*	Yes [112]	Yes [112]	NA
*Thioguanine*	Yes [113]	Yes [113]	WEE1 inhibition enhanced cytotoxicity [65]
*Topotecan*	No	Yes [114]	WEE1 inhibition enhanced cytotoxicity [115]
*Vinblastine*	No	Yes [116]	NA
*Vincristine*	No	Yes [117]	WEE1 inhibition enhanced cytotoxicity [118]

### Preclinical studies of WEE1 and PKMYT1 inhibitors

Several targeted compounds showed an inhibitory activity on WEE1 and PKMYT1 kinases and their efficacy was proven in a number of tumor types. Table 3 shows the main preclinical studies that used WEE1/PKMYT1 inhibitors in single agent or in combination with chemo/radiotherapy agents in different tumor types.

**Table 3:** Preclinical studies evaluating the effect of WEE1 inhibitors in monotherapy or in combination with chemotherapy/radiotherapy in cancer

Inhibitor	Treatment	Cancer model	Main biological effect	References
PD0166285	M	GBM-astrocytoma	-G2/M checkpoint override -Forced mitotic entry	[51]
Adavosertib	M	MM, ALL, AML TNBC, DLBCL, MCL	-G2/M checkpoint override -Forced mitotic entry -Mitotic catastrophe -Replicative catastrophe	[35, 99, 119–122]
PD0166285	+R	GBM-astrocytoma	-Mitotic catastrophe -Inhibition of DNA repair	[51]
Adavosertib	+R	CC, LC, BC, PC, OC, DLBCL, ES	-Increased DNA damage -Induction of apoptosis -Mitotic catastrophe	[78, 99, 123–126]
Adavosertib	+C	AML, ALL, MM, BC, CC, GC, DLBCL	-S or G2/M checkpoint override -Increased DNA damaged -Induction of apoptosis	[35, 37, 38, 76, 92, 99, 121, 127–129]
Adavosertib	+HDAC i	AML, HNSCC	-Replication stress -Replicative catastrophe -Increased DNA damage -Inhibition of DNA repair	[41, 130, 131]
Adavosertib	+ATR i	AML, DLBCL, MCL, BC	-Replication stress -Replicative catastrophe -Increased DNA damaged -Inhibition of DNA repair	[132–135]
Adavosertib	+mTOR i	AML, ALL, OC, NSCLC	-Inhibition of DNA repair	[136–139]
Adavosertib	+CHK1 i	MCL, DLBCL, ALL, AML	-Replication stress -Increased DNA damage -Replicative catastrophe	[103, 140–142]
Adavosertib	+BCL2i/MCL-1 i	DLBCL	-Force mitotic entry -Increase DNA damage -INDUCTION of apoptosis	[143]
Adavosertib	+PARP1 i	NSCLC, AML, ALL	-G2/M checkpoint override -Replication stress -Increased DNA damage -Inhibition of DNA repair	[126, 144–146]
Adavosertib	+AURORA A i	HNSCC	-Forced mitotic entry -Mitotic catastrophe	[147]
Adavosertib	+CDK2 i	BC	-Replication stress -Replicative catastrophe	[89]
Adavosertib	+SIRT1 i	LC	-Inhibition of DNA repair	[148]
Adavosertib	+CDK4-6 i	S	-Replication stress	[149]
Adavosertib	+BCR-ABL1 i	ALL	-Inhibition of DNA repair -G2/M checkpoint override	[35]
Adavosertib	+Proteasome i	MM	-G2/M checkpoint override -Forced mitotic entry -Inhibition of DNA repair	[36]
Adavosertib	+BET i	NSCLC	-Inhibition of DNA repair -Forced mitotic entry -Mitotic catastrophe	[150]

*M* monotherapy, *R* radiotherapy, *C* chemotherapy, *ALL* acute lymphoblastic leukemia, *AML* acute myeloid leukemia, *MM* multiple myeloma, *DLBCL* diffuse large B cell lymphoma, *MCL* mantle cell lymphoma, *GC* gastric cancer, *GL* gliomas, *OC* ovarian cancer, *CC* colorectal cancer, *PC* pancreatic cancer, *ES* esophageal cancer, *HC* hepatocellular carcinoma, *GLB* glioblastoma, *NSCLC* non-small-cell lung cancer, *N* neuroblastoma, *S* sarcomas, *LC* lung cancer, *BC* breast cancer, *HNSCC* head and neck squamous cell carcinoma, *TNBC* triple negative breast cancer

PD0166285 is the first reported drug, with an inhibitory activity against WEE1, PKMYT1, and a range of other kinases including c-Src, EGFR, FGFR1, CHK1, and PDGFRb [151].

Adavosertib (AZD-1775) is the first highly potent and selective WEE1 inhibitor. A large number of preclinical studies evaluated its efficacy in single agent and in combinatory approaches. Regarding the mechanism of action, adavosertib induces S and/or G2/M cell cycle checkpoints override, depending on cancer types, when used in monotherapy. Cell cycle perturbation is associated with a progressive accumulation of DNA damages and by the induction of apoptosis [35, 99, 119–122]. This last event is cell cycle phase-dependent and can occur (i) as a consequence of S phase checkpoint override, when cancer cells start DNA replication even in the presence of DNA damages (replicative catastrophe); (ii) following G2/M phase checkpoint override, that results in forced entry into mitosis, even in the presence of DNA damages (mitotic catastrophe).

In combination strategies, adavosertib was able to enhance the cytotoxicity of chemo/radiotherapy agents, by inducing cell cycle checkpoint override, inhibition of DNA damage repair, and induction of apoptosis [35, 37, 38, 92, 121, 127–129]. The chemo-sensitizer efficacy of DDR inhibitors has been linked to drug scheduling [94, 152, 153]. Recently in pancreatic adenocarcinoma cells, it has been reported that the efficacy of a triple regimen combining gemcitabine, CHK1, and WEE1 inhibitors is strictly dependent on the timing of drug administration. Indeed, the maximum effect of the combination is obtained when gemcitabine and CHK1 inhibitors are administered simultaneously (thus inducing replicative stress) and adavosertib is added at a later time [94].

Moreover, strong synergism has been observed by combining adavosertib with small molecules, including DDR-related inhibitors (CDK2 [89], CDK4-6 [149], CHK1 [103, 140–142], ATM [132–135], AURORA A [147], PARP1 [144], SIRT1 [148] inhibitors), histone deacetylase (HDAC) inhibitors [41, 130, 131], tyrosine kinase inhibitors (BCR-ABL1 inhibitors [35]), anti-apoptotic protein inhibitors (BCL2 and MCL1 inhibitors [143]), mTOR inhibitor [136–139], and proteasome inhibitors [36].

We have recently reported synergistic effects of adavosertib in combination with different tyrosine kinase inhibitors in both BCR-ABL1-positive and -negative ALL cell lines and primary cells. Interestingly, strong synergism was found in *BCR*-*ABL1*-negative ALL cell lines treated with adavosertib in combination with bosutinib isomer. In the study, we speculated that the strong cytotoxic effect of the combination was due to the concomitant inhibition of WEE1 and PKMYT1 kinases [35]. Indeed, no selective inhibitor has been currently developed to target its functionality. However, several known tyrosine kinase inhibitors have an inhibitory off-target effect on PKMYT1. Among them, compounds commonly used for the treatment of *BCR-ABL1*-positive CML and ALL, as dasatinib and bosutinib (and a structural isomer of bosutinib [154, 155]) were shown to inhibit PKMYT1 activity.

Overall, the data suggest that WEE1/PKMYT1 inhibition is a suitable pharmacological target for combination strategies in cancer. The broad spectrum of activities exerted by the two kinases, and especially by WEE1, across the cell cycle, makes them good candidates for a number of diverse therapeutic combinations.

### WEE1 inhibitors from bench to bedside

Several clinical studies are currently evaluating the efficacy of adavosertib on different aggressive and advanced tumors (Table 4).

**Table 4:** Clinical trials evaluating WEE1/PKMYT1 inhibitor in monotherapy or in combination for cancer therapy

Study ID	Study title	Tumor	Interventions	Status	Phase
NCT02610075	Phase Ib Study to Determine MTD of AZD1775 Monotherapy in Patients With Locally Advanced or Metastatic Solid Tumours.	S	AZD1775	C	1
NCT03668340	AZD1775 in Women With Recurrent or Persistent Uterine Serous Carcinoma	S	AZD1775	R	2
NCT02482311	Safety, Tolerance, PK, and Anti-tumour Activity of AZD1775 Monotherapy in Patients With Advanced Solid Tumours	S	AZD 1775	C	1
NCT02207010	A Phase 0 Study of AZD1775 in Recurrent GBM Patients	S	AZD1775	NA	1
NCT03315091	Phase I Study to Assess the Effect of Food on AZD1775 Pharmacokinetics in Patients With Advanced Solid Tumours	S	AZD1775	C	1
NCT01748825	AZD1775 for Advanced Solid Tumors	S/H	AZD1775	ANR	1
NCT02511795	AZD1775 Combined With Olaparib in Patients With Refractory Solid Tumors	S	AZD1775 + Olaparib	C	1
NCT03313557	AZD1775 Continued Access Study to Assess Safety and Tolerability for Patients Enrolled in AZD1775 Clinical Pharmacology Studies	S	AZD1775	C	1
NCT02593019	Phase II, Single-arm Study of AZD1775 Monotherapy in Relapsed Small Cell Lung Cancer Patients	S	AZD1775	NA	2
NCT02688907	Phase II, Single-arm Study of AZD1775 Monotherapy in Relapsed Small Cell Lung Cancer Patients With MYC Family Amplification or CDKN2A Mutation Combined With TP53 Mutation	S	AZD1775	T	2
NCT02087176	A Placebo Controlled Study Comparing AZD1775 + Docetaxel Versus Placebo + Docetaxel to Treat Lung Cancer	S	AZD1775 + Docetaxel	T	2
NCT03012477	CISPLATIN + AZD-1775 In Breast Cancer	S	AZD1775 + Cisplatin	ANR	2
NCT02341456	Phase Ib Study AZD1775 in Combination With Carboplatin and Paclitaxel in Adult Asian Patients With Solid Tumours	S	AZD1775 + Carboplatin or Paclitaxel	C	1
NCT02791919	Wee1 Kinase Inhibitor AZD1775 and Combination Chemotherapy in Treating Children, Adolescents and Young Adults With Relapsed or Refractory Acute Myeloid Leukemia	H	AZD1775 + Cytarabine or Filgrastim or Fludarabine Phosphate	W	1
NCT02513563	AZD1775 Plus Carboplatin-Paclitaxel in Squamous Cell Lung Cancer	S	AZD1775 + Carboplatin or Paclitaxel	R	2
NCT03718143	AZD1775 in Advanced Acute Myeloid Leukemia, Myelodysplastic Syndrome and Myelofibrosis	H	AZD1775 + Cytarabine	T	2
NCT02585973	Dose-escalating AZD1775 + Concurrent Radiation + Cisplatin for Intermediate/High Risk HNSCC	S	AZD1775 + Cisplatin + Radiation	R	1
NCT02087241	Ph II Trial of Carboplatin and Pemetrexed With or Without AZD1775 for Untreated Lung Cancer	S	AZD1775 + pemetrexed or carboplatin	T	2
NCT02381548	Phase I Trial of AZD1775 and Belinostat in Treating Patients With Relapsed or Refractory Myeloid Malignancies or Untreated Acute Myeloid Leukemia	H	AZD1775 + Belinostat	T	1
NCT03333824	Effects of AZD1775 on the PK Substrates for CYP3A, CYP2C19, CYP1A2 and on QT Interval in Patients With Advanced Cancer	S	AZD1775	C	1
NCT02906059	Study of Irinotecan and AZD1775, a Selective Wee 1 Inhibitor, in RAS or BRAF Mutated, Second-line Metastatic Colorectal Cancer	S	AZD1775 + Irinotecan	R	1
NCT02037230	Dose Escalation Trial of AZD1775 and Gemcitabine (+Radiation) for Unresectable Adenocarcinoma of the Pancreas	S	AZD1775 + Gemcitabine+ Radiation Therapy	C	1,2
NCT02617277	Safety, Tolerability and Pharmacokinetics of AZD1775 (Adavosertib) Plus MEDI4736 (Durvalumab) in Patients With Advanced Solid Tumours	S	AZD1775 + Durvalumab	ANR	1
NCT02666950	WEE1 Inhibitor AZD1775 With or Without Cytarabine in Treating Patients With Advanced Acute Myeloid Leukemia or Myelodysplastic Syndrome	H	AZD1775 + Cytarabine	C	2
NCT01047007	A Dose Escalation Study of MK1775 in Combination With 5-FU or 5-FU/CDDP in Patients With Advanced Solid Tumor (1775-005)	S	AZD1775 + 5-FU or 5-FU/CDDP	T	1
NCT01164995	Study With Wee-1 Inhibitor MK-1775 and Carboplatin to Treat p53 Mutated Refractory and Resistant Ovarian Cancer	S	AZD1775 + carboplatin	NA	2
NCT02448329	Study of AZD1775 in Combination With Paclitaxel, in Advanced Gastric Adenocarcinoma Patients Harboring TP53 Mutation as a Second-line Chemotherapy	S	AZD1775 + paclitaxel	R	2
NCT02508246	WEE1 Inhibitor MK-1775, Docetaxel, and Cisplatin Before Surgery in Treating Patients With Borderline Resectable Stage III-IVB Squamous Cell Carcinoma of the Head and Neck	S	AZD1775 + Cisplatin + Docetaxel	C	1
NCT03253679	AZD1775 in Treating Patients With Advanced Refractory Solid Tumors With CCNE1 Amplification	S	AZD1775	R	2
NCT01076400	A Study of MK-1775 in Combination With Topotecan/Cisplatin in Participants With Cervical Cancer (MK-1775-008)	S	AZD1775 + Topotecan or Cisplatin	T	1,2
NCT02196168	Cisplatin With or Without WEE1 Inhibitor MK-1775 in Treating Patients With Recurrent or Metastatic Head and Neck Cancer	S	AZD1775 +Cisplatin	T	2
NCT02101775	Gemcitabine Hydrochloride With or Without WEE1 Inhibitor MK-1775 in Treating Patients With Recurrent Ovarian, Primary Peritoneal, or Fallopian Tube Cancer	S	AZD1775 + Gemcitabine	ANR	2
NCT03028766	WEE1 Inhibitor With Cisplatin and Radiotherapy: A Trial in Head and Neck Cancer	S	AZD1775 + Cisplatin + Radio therapy	ANR	1
NCT01357161	A Study of MK-1775 in Combination With Paclitaxel and Carboplatin Versus Paclitaxel and Carboplatin Alone for Participants With Platinum-Sensitive Ovarian Tumors With the P53 Gene Mutation (MK-1775-004)	S	AZD1775 + paclitaxel + carboplation	C	2
NCT03284385	Testing AZD1775 in Advanced Solid Tumors That Have a Mutation Called SETD2	S	AZD1775	R	2
NCT00648648	A Dose Escalation Study of MK-1775 in Combination With Either Gemcitabine, Cisplatin, or Carboplatin in Adults With Advanced Solid Tumors (MK-1775-001)	S	AZD1775 + Gemcitabine or Cisplatin or Carboplatin	C	1
NCT02194829	Paclitaxel Albumin-Stabilized Nanoparticle Formulation and Gemcitabine Hydrochloride With or Without WEE1 Inhibitor MK-1775 in Treating Patients With Previously Untreated Pancreatic Cancer That Is Metastatic or Cannot Be Removed by Surgery	S	AZD-1775 + Gemcitabine + paclitaxel	ANR	1,2
NCT02576444	Olaparib Combinations	S	AZD1775 + olaparib	ANR	2
NCT04197713	Testing the Sequential Combination of the Anti-cancer Drugs Olaparib Followed by Adavosertib (AZD1775) in Patients With Advanced Solid Tumors With Selected Mutations and PARP Resistance, STAR Study	S	AZD1775 + olaparib	ANR	1
NCT01922076	Adavosertib and Local Radiation Therapy in Treating Children With Newly Diagnosed Diffuse Intrinsic Pontine Gliomas	S	AZD1775 + Radiation Therapy	ANR	1
NCT03579316	Adavosertib With or Without Olaparib in Treating Patients With Recurrent Ovarian, Primary Peritoneal, or Fallopian Tube Cancer	S	AZD1775 + olaparib	R	2
NCT02095132	Adavosertib and Irinotecan Hydrochloride in Treating Younger Patients With Relapsed or Refractory Solid Tumors	S	AZD1775 + Irinotecan or Irinotecan Hydrochloride	R	1,2
NCT03345784	Adavosertib, External Beam Radiation Therapy, and Cisplatin in Treating Patients With Cervical, Vaginal, or Uterine Cancer	S	AZD1775 +Cisplatin + Radiation (External Beam Radiation Therapy)	R	1
NCT01849146	Adavosertib, Radiation Therapy, and Temozolomide in Treating Patients With Newly Diagnosed or Recurrent Glioblastoma	S	AZD1775 + Radiation Therapy + Temozolomide	R	1
NCT02937818	A Phase II, Study to Determine the Preliminary Efficacy of Novel Combinations of Treatment in Patients With Platinum Refractory Extensive-Stage Small-Cell Lung Cancer	S	AZD1775 + carboplatin	ANR	2
NCT02546661	Open-Label, Randomised, Multi-Drug, Biomarker-Directed, Phase 1b Study in Pts w/ Muscle Invasive Bladder Cancer	S	AZD1775 + Durvalumab	ANR	1
NCT02659241	Adavosertib Before Surgery in Treating Patients With Advanced High Grade Ovarian, Fallopian Tube, or Primary Peritoneal Cancer	S	AZD1775	R	1
NCT02272790	Adavosertib Plus Chemotherapy in Platinum-Resistant Epithelial Ovarian, Fallopian Tube, or Primary Peritoneal Cancer	S	AZD1775 + Paclitaxel or Carboplatin or Gemcitabine or pegylated liposomal doxorubicin	ANR	2
NCT02813135	European Proof-of-Concept Therapeutic Stratification Trial of Molecular Anomalies in Relapsed or Refractory Tumors	S/H	AZD1775 + carboplatin	R	1,2
NCT03330847	To Assess Safety and Efficacy of Agents Targeting DNA Damage Repair With Olaparib Versus Olaparib Monotherapy.	S	AZD1775 + olaparib	R	2
NCT01827384	MPACT Study to Compare Effects of Targeted Drugs on Tumor Gene Variations	S	AZD1775 + carboplatin	R	2
NCT02465060	Targeted Therapy Directed by Genetic Testing in Treating Patients With Advanced Refractory Solid Tumors, Lymphomas, or Multiple Myeloma (The MATCH Screening Trial)	S/H	AZD1775	R	2

*S* solid tumor, *H* hematological tumor, *C* completed, *R* recruiting, *W* withdraw, *ANR* active not recruiting, *T* terminated, *NA* status unknown (last update 04/22/2020)

The results of phase I trials showed that adavosertib is well tolerated both in single agent and in combination. Depending on the study, the maximum tolerated dose (MTD) was established between 150 and 225 mg orally twice per day for 2.5 days per 2 weeks [156–158]. The most common adverse events reported in the abovementioned studies were fatigue, nausea, vomiting, diarrhea, and hematologic toxicity. Moreover, correlative studies performed on tumor biopsies confirmed in vivo the mechanism of action of adavosertib. Indeed, immunohistochemistry analyses showed a reduction of phospho-CDK1 (Tyr15) and an increase of DNA damages (phospho-γH2AX) in cancer cells [156, 157].

The phase II studies confirmed that adavosertib sensitizes cancer patients to different chemotherapy agents. Interestingly, adavosertib showed efficacy when combined with carboplatin in *TP53*-mutated ovarian cancer patients, refractory or resistant to first-line platinum-based chemotherapy [159]. Similar results were reported in platinum-resistant primary ovarian cancer patients after treatment with the combination of adavosertib and a single chemotherapeutic agent (carboplatin, paclitaxel, gemcitabine, or pegylated liposomal doxorubicin) [160].

## Primary resistance and predictive markers of response to WEE1/PKMYT1 based therapies

Several DDR inhibitors have proved their efficacy against different cancer types in the preclinical and clinical settings [161–165]. Among them, WEE1 inhibitor seems to be the most effective ones, also favored by a relative low off-target toxicity. However, despite the number of studies and the promising results, few predictive markers of response have been identified. Recently, cyclin E level has been linked to the efficacy of adavosertib in breast cancer models [89], with cyclin E-high cells, that generally show elevated chromosome instability, being more sensitive compared with cyclin E-low ones. Despite the reported low levels of *WEE1* expression in breast cancer, chromosome instability, that has also prognostic potential mainly in grade 2 tumors [89], may explain the effectiveness of WEE1 inhibitors, as supported by the predictive role of cyclin E. Our group and others showed that high PKMYT1 expression associates with reduced sensitivity to adavosertib, indicating a potential compensatory effect [35, 166]. Moreover, high-throughput proteomic profiling demonstrated that small cell lung cancer and ovarian cancer models with primary resistance to adavosertib express high levels of AKT/mTOR pathway molecules and phosphorylated S6 ribosomal protein [137, 138]. In acute leukemia models, the sensitivity to adavosertib has been recently linked to HDAC and MYC regulation. Indeed, by generating adavosertib-resistant models, the researchers found that resistant acute leukemia cell lines are dependent on increased HDAC activity for their survival, partly due to increased KDM5A function. In addition, gene expression analyses demonstrated a HDAC-dependent expression of MYC in the adavosertib-resistant cell lines [167]. These observations support the success of preclinical studies combining WEE1 and HDAC [41, 130, 131] or bromodomain inhibitors [150].

## Conclusion

Thanks to a constantly growing amount of preclinical and clinical data, our knowledge on cancer biology is increasing and, consequently, the list of cancer hallmarks has been progressively expanding. Recent findings demonstrated that cancer cells are characterized by functional and molecular alterations in crucial genes involved in the DDR pathway, which is fundamental for cell cycle regulation, DNA damages recognition, and repair. Functional alterations of DDR-gene have a deep impact on tumor progression and on the clinical outcome of cancer patients. Indeed, the efficacy of standard of care chemo/radiotherapy regimens depends on the generation of DNA damages in proliferating malignant cells. In this scenario, the overexpression or uncontrolled activation of DDR pathways has been showed to protect cancer cells from the therapeutic effect of DNA damaging agents. Moreover, a large number of preclinical studies highlighted that cancer cells depend on the functionality of DDR pathways in order to survive, to tolerate the replicative stress induced by the high proliferative rate and to sustain the intrinsic genetic instability. For these reasons, selective inhibitors have been developed in order to exploit cancer cells’ dependency on DDR-gene functionality. Pre-clinical data has proven the efficacy of DDR inhibition in different kinds of hematological and solid tumors, both as monotherapy and in combination with a wide number of DNA damaging agents. Among DDR inhibitors, the most effective once are those targeting PARP1 and WEE1 family kinases. The effectiveness of PARP1 inhibitors is however dependent on homologous recombination (HR) repair deficiency while WEE1 family kinases inhibitors seems to have a widespread efficacy independently from a specific the genetic background. Indeed, cancer cells seem to be strictly dependent on the functionality of WEE1/PKMYT1 kinases to survive, especially those with alterations targeting the G1 checkpoint. WEE1/PKMYT1 kinases are involved in different biological processes and they seem to play diverse roles in nonmalignant and in cancer cells. Indeed, they control cell cycle regulation and genetic stability in nonmalignant cells and for these reasons act as tumor suppressor genes. Conversely, their ability of promote DNA damages repair and cell cycle control makes them act as pseudo-oncogenes in cancer cells. Several molecular studies showed that malignant cells have high expression level of *WEE1* and *PKMYT1*, which has become a good prognostic biomarker for chemo/radiotherapy regimens. However, we currently lack information regarding predictive markers of response to WEE1/PKMYT1 inhibitors. Large preclinical and clinical studies should be conducted in order to identify specific molecular backgrounds in which the use of WEE1/PKYMT1 inhibitors may be recommended. The identification of molecular vulnerabilities in cancer patients will be fundamental to design novel therapeutic regimens using WEE1/PKMYT1 inhibitors in a chemo/radiotherapy-free, synthetic lethality-based approach.

## Data Availability

Not applicable.

## Abbreviations

ALL: Acute lymphoblastic leukemia
AML: Acute myeloid leukemia
BC: Breast cancer
C: Chemotherapy
CC: Colorectal cancer
CN: Copy number
CNA: Copy number alteration
CLL: Chronic lymphocyte leukemia
CML: Chronic myeloid leukemia
DDR: DNA damage response
DLBCL: Diffuse large B cell lymphoma
ES: Esophageal cancer
GC: Gastric cancer
GL: Gliomas
GLB: Glioblastoma
HC: Hepatocellular carcinoma
HR: Homologous recombination
HNSCC: Head and neck squamous cell carcinoma
LC: Lung cancer
M: Monotherapy
MaM: Malignant melanoma
MCL: Mantle cell lymphoma
MM: Multiple myeloma
MPF: Mitotic promoting factor
N: Neuroblastoma
NSCLC: Non-small-cell lung cancer
OC: Ovarian cancer
PC: Pancreatic cancer
R: Radiotherapy
S: Sarcomas
SAC: Spindle assembly checkpoint
TNBC: Triple-negative breast cancer
